# Spotlight on Secondary Metabolites Produced by an Early-Flowering Apulian Artichoke Ecotype Sanitized from Virus Infection by Meristem-Tip-Culture and Thermotherapy

**DOI:** 10.3390/antiox13070852

**Published:** 2024-07-16

**Authors:** Roberta Spanò, Patrizia Gena, Vito Linsalata, Valeria Sini, Isabella D’Antuono, Angela Cardinali, Pietro Cotugno, Giuseppe Calamita, Tiziana Mascia

**Affiliations:** 1 Department of Soil, Plant and Food Sciences, University of Bari “Aldo Moro”, Via Amendola 165/A, 70126 Bari, Italy; tiziana.mascia@uniba.it; 2 Department of Biosciences, Biotechnologies and Environment, University of Bari “Aldo Moro”, Via E. Orabona 4, 70125 Bari, Italy; annapatrizia.gena@uniba.it (P.G.); valeria.sini@uniba.it (V.S.); giuseppe.calamita@uniba.it (G.C.); 3 Institute of Science of Foods Production (ISPA)–CNR Via Amendola 122/O, 70126 Bari, Italy; vito.linsalata@ispa.cnr.it (V.L.); isabella.dantuono@ispa.cnr.it (I.D.); angela.cardinali@ispa.cnr.it (A.C.); 4 Department of Chemistry, University of Bari “Aldo Moro”, Via Orabona 4, 70125 Bari, Italy; pietro.cotugno@uniba.it

**Keywords:** artichoke, antioxidant and antimicrobial effects, anti-inflammatory effects, polyphenol extracts, supercritical fluid extraction, RNAseq analysis, virus infection, virus sanitation protocol

## Abstract

Globe artichoke (*Cynara cardunculus* L. subsp. *scolymus*) is an important crop of the Mediterranean basin characterized by many properties, like hepatoprotective, anticarcinogenic, antioxidant, antibacterial, and beneficial to human health. The high bioactive compounds (BACs) content, as polyphenols, has attracted the research interest in artichoke extracts. We analysed the changes in polyphenol transcriptome profile between sanitized (S) virus-free and non-sanitized (NS) artichoke plants, focusing on genes involved in phenylpropanoid metabolic pathway and flavonoid biosynthesis. A total of 2458 upregulated and 2154 downregulated differentially expressed genes (DEGs) were functionally characterized. Among them, 31 and 35 KEGG orthology entries characterized by upregulated and downregulated DEGs, respectively, were involved in the biosynthesis of other secondary metabolites. A downregulation of *PAL*, *C4H*, *4CL*, *HST/HQT*, *C3′H*, *CCoAMT*, *CCR1,* and *F5H*, was observed in S artichoke compared to NS one, whereas the *CSE*, *CHS*, and *CHI* genes were upregulated in S samples. Transcriptome results were compared to the polyphenols accumulation in S and NS artichoke leaves. A higher content of total polyphenols was observed in older leaves of NS samples, compared to extracts obtained from young leaves or from S plants, and this result was associated with the presence of viral infections in NS plants. In all the conditions tested, the most represented compound was chlorogenic acid, followed by luteolin-7-*O*-glucoside. The different composition of each extract was evaluated by a polyphenol dose–response treatment on the rodent hepatoma FaO cell line to the accumulation of reactive oxygen species (ROS). A significant reduction in ROS content ranging between −40% and −48% was observed when 10–20 mg/L of polyphenols from NS or S plants were used, characterized by a specific profile of compounds. To reduce MetOH residues in polyphenol extracts, a supercritical fluid CO_2_ extraction was evaluated to propose a sustainable green extraction.

## 1. Introduction

Globe artichoke (*Cynara cardunculus* L. subsp. *scolymus*) belongs to the *Asteraceae* family. Known since the 4th century B.C. as a food and remedy, globe artichoke is an ancient crop of the Mediterranean basin, cultivated by Greeks and Romans and later introduced to other parts of Europe. Today, artichokes are grown in several countries around the world, with Italy, Spain, and France accounting as the largest producers. The domestication process led to the selection of varieties based on the precocity of the productions, dimension, and absence of spines in the immature inflorescences, which are called capitula or heads. In the production area, new ecotypes were also selected based on agronomic performance, like the ‘Brindisino’ (BR) artichoke ecotype. The morphological characteristics of this ecotype are a medium height size with a high offshoot production and green leaves with high heterophylly. The vegetative cycle is from July to June, producing capitula between autumn and early spring, thus denoted early-flowering artichoke. The immature flower head has a cylindrical shape, medium compact, with a minimum height of 8 cm and a minimum diameter of 6 cm. External bracts are green with violet shades, whole or slightly incised rounded apex, which may have a little thorn; internal bracts are greenish white with slight violet shades; stem is thin or medium thickness of no more than 10 cm. The disciplinary production referred to as this ecotype is obtained only in specific districts of the Brindisi province (Apulia, southern Italy). These plants are labelled as “Protected Geographical Indication” (PGI) products, based on the Regulation (CE) n. 510/2006 and Directive 447-03/08/12 of the Apulian Regional Government.

Notably, artichokes are particularly rich in bioactive compounds (BACs), like inulin, fibres, minerals, and polyphenols, which are found in the capitula but also at higher levels in leaves. These compounds have applications in the nutraceutical and pharmaceutical industries due to the following health-benefiting properties: digestibility, lipid-lowering, diuretic, mineral-binding capacity [1,2,3], choleretic, anticarcinogenic, anti-HIV, antioxidative, antifungal and antibacterial [4,5]. In addition, polyphenols have a strong anti-age effect due to their anti-free radical ability and UV protection [4]. However, a wide range of biotic and abiotic stresses, such as pathogens infection, insect attacks, light intensity, water deficiency, fertilization, and phenological stage of the plant, as well as the genotype, may deeply affect the BACs profile [5,6,7].

While many biotic and abiotic stressors may affect globe artichoke, plant virus infections are particularly detrimental to crop production and overall quality, including the BAC content. To limit the latter effects, the BR and a number of other global artichoke ecotypes relevant to Apulian production have been sanitized from viral infections by means of meristem-tip-culture and thermotherapy [8]. However, the effects of virus infection and sanitation protocol on BAC accumulation and profile in BR are unknown.

Synthesis of polyphenols in plants depends on the activity of many gene products modulated in response to external factors and the intrinsic needs of the plant. The phenylpropanoid pathway is the starting point for the synthesis of all metabolites containing the phenylpropane skeleton, like polyphenols, flavonoids, and lignin. The regulation of rate-limiting enzymes such as the phenylalanine ammonia-lyase (PAL), chalcone synthase (CHS), and chalcone isomerase (CHI) by transcription factors (e.g., MYB, bHLH, and WD40), which bind to the promoters of these genes, controls the accumulation of polyphenols and other secondary metabolites [9], like flavonoids [10,11].

In this study, we conducted a transcriptome analysis to identify the key genes related to polyphenols accumulation in BR plants certified as virus-free artichoke sanitized (S) by in vitro meristem-tip-culture combined with a thermotherapy treatment [8]. The influence of sanitation protocol on the content of BACs in artichoke has only recently been investigated on two late-flowering ecotypes (producing capitula during spring and early summer) [12]. In the previous study, phytochemical analysis showed a remarkable decrease in polyphenols and lignin accumulation in S artichokes compared to non-sanitized (NS) plants, associated with the upregulation of genes involved in the biosynthesis of secondary metabolites. In late-flowering ecotypes like locale di Mola tardivo (LM) and Troianella (TR), the different phytosanitary status S vs. NS and the genotype modulate the plant response to polyphenols accumulation. This study deals with an in-depth analysis of the early-flowering BR ecotype to better understand the key factors of polyphenol production and accumulation in S vs. NS artichoke plants and to promote the use of this autochthonous ecotype for its high health-promoting compound.

The beneficial properties of artichoke polyphenols are mainly derived from chlorogenic acid and dicaffeoylquinic acids but also from their combination with other minor polyphenol derivatives. Moreover, their content changes in different portions of the plant [5], which, in turn, may have effects on the antioxidant capacity of raw material if artichoke crop biomass is used for BAC extraction. Thus, we investigated the biological activity of the artichoke polyphenol profile extracted from S and NS BR plants and from different tissues by measuring their effects on cell viability and for potential use as nutraceutical compounds.

To our knowledge, this is the first detailed investigation of polyphenol biosynthesis at molecular, chemical, and biological levels in a sanitized early-flowering artichoke ecotype.

## 2. Materials and Methods

### 2.1. Plant Materials and Phytosanitary Status

Young artichoke offshoots of 10–15 cm in length were collected in late September 2021 from S and NS BR ecotypes. S plants were obtained by in vitro meristem-tip-culture combined with a thermotherapy treatment, certified as virus-free primary source germplasm, and conserved in a commercial nursery (Vivaio F.lli Corrado, Torre Santa Susanna, Brindisi, Apulia, southern Italy). NS plants were grown in natural conditions in open-field crops located in Brindisi province (Apulia, southern Italy).

To avoid biases introduced by the operator, a systematic random sampling design [13] was used to select young artichoke offshoots (10–15 cm in length) of ten NS stocks, from a commercial open-field crop and of ten S stocks from the primary source germplasm. To diminish biases introduced by the different origin (open-field or commercial nursery), all the collected offshoots were transplanted into 18 cm diameter pots (3.5 L) containing a substrate of brown and white peat in a ratio of 1:4, mixed with expanded clay. Plants were maintained ex situ in a greenhouse at a temperature (T) of 18–20 °C, 55–60% relative humidity (RH), and a 16 h light/8 h dark photoperiod and used for further experiments. Plants developed from such offshoots were considered biological replicates of the NS and S phytosanitary conditions and are hereafter denoted as BR-NS and BR-S plants.

### 2.2. Total RNA Purification and Sequencing

Total RNA was purified from 100 mg of fresh leaf tissue of BR-S and BR-NS samples (in triplicate) ground in liquid nitrogen and extracted following the EuroGOLD RNAPure^TM^ (EuroClone, Pero, Italy) protocol. RNA concentration, quality, and integrity were analyzed by Qubit RNA HS assay kit (ThermoFisher Scientific, Waltham, MA, USA), Bioanalyzer, RNA 6000 Pico Labchip (Agilent Technologies, Santa Clara, CA, USA), and agarose gel electrophoresis. Extracts with an RNA integrity number (RIN) ≥ 7 were used for virus detection by the polyprobe hybridization assay [14] and RNA sequencing (RNAseq) library preparation.

RNA samples were ribo-depleted and reverse transcribed prior to sequencing on an Illumina HiSeq 2 × 150 bp reads platform (GENEWIZ, Azenta Life Sciences, Leipzig, Germany).

### 2.3. Transcriptome Data Analysis

Sequencing data were analyzed using Galaxy platform (https://usegalaxy.eu, accessed on 17 April 2023) tools as described by Spanò et al. [8], using the *C. cardunculus* sequence (Acc. N. GCA_001531365.1) as reference genome.

Sequence alignments were also performed against viruses detected by the hybridization assay using reads unmapped on the *C. cardunculus* genome. According to the list of virus species recognized as detrimental to global artichoke production by Directive 447-03/08/12 of the Apulian Regional Government. The virus genome sequences used in the Bowtie2 alignment tool [15,16] were artichoke Italian latent virus (AILV) (GenBank Acc. N. LT608395.1 and LT608396.1 for RNA1 and RNA2, respectively), artichoke mottled crinkle virus (AMCV) (GeneBank Acc. N. NC_001339.1), artichoke latent virus (ArLV) (GeneBank Acc. N. KF155694.1), bean yellow mosaic virus (BYMV) (GeneBank Acc. N. HG970854.1), cucumber mosaic virus (CMV) (GeneBank Acc. N. NC_002034.1, NC_002035.1, and NC_001440.1 for RNA1, RNA2, and RNA3, respectively), pelargonium zonate spot virus (PZSV) (GeneBank Acc. N. AJ272327.1, AJ272328.2, and AJ272329.1 for RNA1, RNA2, and RNA3, respectively), tomato infectious chlorosis virus (TICV) (GeneBank Acc. N. NC_013258.1 and NC_013259.1 for RNA1 and RNA2, respectively), tobacco mosaic virus (TMV) (GeneBank Acc. N. AB369276.1), tomato spotted wilt virus (TSWV) (GeneBank Acc. N. KT717691.1, KT717692.1, and KT717693.1 for RNA1, RNA2, and RNA3, respectively) and turnip mosaic virus (TuMV) (GeneBank Acc. N. AP017717.1). Mapped reads were visualized using Integrative Genomics Viewer (IGV) [17,18], and the presence of virus infection was validated by polymerase chain reactions (PCR) according to Minutillo et al. [14].

Read counts for each gene were calculated using FeatureCounts [19]. Gene expression in the S and NS samples was calculated and normalized to reads per kilobase per million mapped reads (RPKM) [20]. ‘Sanitized (BR-S) vs. naturally virus-infected (BR-NS)’ comparison was set for gene expression analysis using DESeq2 tool [21] with default parameters. Genes were considered significantly differentially expressed (DEGs) in S vs. NS conditions when the logarithm (to basis 2) of the absolute fold change (FC) was ≥1, with a false discovery rate (FDR) ≤ 0.05 [12,22]. Gene ontology (GO) enrichment (*p*-value < 0.05) of upregulated and downregulated DEGs was performed using BlastKOALA tool (https://www.kegg.jp/blastkoala/, accessed on 21 June 2023) [23] to further classify genes into KEGG Orthology (KO) entry and understand their involvement in biological pathways.

### 2.4. Validation of RNAseq Results by qRT-PCR

First-strand cDNA was synthesized from 1 μg of total RNA prepared from S and NS artichoke samples. RNA was pre-treated with a TURBO DNA-free kit (ThermoFisher Scientific, Waltham, MA, USA) and reverse transcribed with random hexamers using the Tetro cDNA synthesis kit (Bioline Reagents Ltd., London, United Kingdom) according to the manufacturer’s instructions. To estimate the relative abundance of genes, the comparative cycle threshold (2^−ΔΔCt^) method normalized for polymerase chain reaction (PCR) efficiencies was used, using *elongation factor 1 alpha* (*EF-1a*) as a housekeeping gene for target gene normalization [12,24]. Quantitative real-time PCR (qPCR) was carried out on a StepOne Real-Time PCR system (Applied Biosystems, Waltham, MA, USA) apparatus using 1× PowerUp Sybr Green Master Mix (Applied Biosystems, Life Technologies, Carlsbad, CA, USA) containing 15 ng of first-strand cDNA template, as previously reported [12]. Primer sequences of gene transcript involved in the biosynthesis of secondary metabolites are shown in Table S1.

### 2.5. Artichoke Leaf Extracts and Chemical Analysis

Six plants of each BR-S and BR-NS condition were used for polyphenol extraction by the standard extraction procedure described by D’Antuono et al. [25]. In detail, from each plant, three samples divided into young leaf (YL), intermediate leaf (IL), and old leaf (OF) were collected by weekly sampling during May 2022. Leaves were immediately freeze-dried, and a weight equivalent to 10 g of fresh material was extracted in a water bath at 100 °C in a reflux extractor for 1 h with 100 mL of 50% MetOH (fresh/solvent 1:10 *w*/*v*). Further, the extract was filtered by Whatman 1 filter paper and an aliquot filtered at 0.45 mm with regenerated cellulose filters before the high-pressure liquid chromatography with diode array detection (HPLC-DAD) analysis.

Artichoke polyphenols were also obtained from BR-S leaves by CO_2_ supercritical fluid extraction (SFE) as a green extraction procedure using an Applied Separations apparatus (Hamilton Street, Allentown, PA, USA) for large-scale metabolite production. In particular, extracts were obtained from 15 g of freeze-dried BR-S artichoke leaves corresponding to 130 g of fresh leaf material loaded in a 50 mL pressure vessel. Extraction parameters were 50 °C temperature (T), 300 bar pressure (P), and 4 L/min CO_2_ flow rate in the presence of the co-solvent ethanol (EtOH) at two different concentrations, 15% and 30%, for a total extraction time of 30 min.

Chemical analysis of artichoke leaf extracts obtained by standard extraction or green extraction procedures was performed following the protocol previously described by Spanò et al. [12]. In detail, chemical analysis of leaf extracts was performed by HPLC-DAD on an Agilent 1260 Infinity system (Agilent Technologies, Santa Clara, CA, USA) equipped with a 1260 binary pump, 1260 HiP degasser, 1260 TCC thermostat, 1260 diode array detector, and Agilent Open Lab Chem Station Rev C.01.05 (v.35) software (Agilent Technologies, Santa Clara, CA, USA). The UV-visible absorption chromatogram was detected at 280 nm, 325 nm, and 360 nm. The separation was performed on a 4.6 × 250 mm reversed-phase Luna C-18 (5 μm) column (Phenomenex, Torrance, CA, USA) by gradient elution using methanol (MetOH, eluent A) and water/acetic acid 95:5 (eluent B), according to D’Antuono et al. [25] and as previously described by Spanò et al. [12]. The phenolic compounds were identified and quantified in µg/mL by the retention time, spectra, and response factors of the pure standards. In particular, for the identification were used chlorogenic acid, 3-*O*-caffeoylquinic acid, cynarin, caffeic acid, coumaric acid, 1,5-*O*-di-caffeoylquinic acid, 3,4-*O*-dicaffeoylquinic acid, 3,5-*O*-dicaffeoylquinic acid and 4,5-*O*-di-caffeoylquinic acid, apigenin-7-*O*-glucoside, luteolin, and luteolin-7-*O*-glucoside. All listed standards were supplied by PhytoLab GmbH & Co. KG (Dutendorfer Str. 5-7, 91487 Vestenbergsgreuth Germany). The total polyphenol content was determined as the sum of the individual polyphenols identified by HPLC-DAD.

### 2.6. Cytotoxicity Assay and Reactive Oxygen Species (ROS) Detection

The cytotoxicity of artichoke leaf extracts was evaluated by enzymatic reduction of 3-[4,5-dimethylthiazole-2-yl]-2,5-diphenyltetrazolium bromide (MTT) to MTT-formazan assay according to the manufacturer’s instructions (Biotium, Fremont, CA, USA).

Briefly, rat hepatoma FaO cells [The European Collection of Authenticated Cell Cultures (EACC)] were plated at a density of 10,000 cells per well in Coon’s modified Ham’s F12 medium supplemented with 10% fetal bovine serum (FBS). FaO cells were cultured for 24–48 h at 37 °C in a humidified 5% CO_2_ incubator to reach the optimal density and then exposed to different concentrations of polyphenols extracted from artichoke leaves for 30 min at 37 °C. To evaluate the dose–response, by MTT assay, methanol (used as a vehicle) solutions, and polyphenolic extracts were at first diluted 1/10 (*v*/*v*), starting from which serial dilutions (1/2, *v*/*v*) were tested.

For the MTT assay, cells were incubated for 4 h at 37 °C with 0.8 mg/mL MTT in culture medium. Then, DMSO was directly added into the medium in each well and shaken for 10 min to dissolve the formazan salt. Absorbance was recorded spectrophotometrically at 570 nm using a Varian Cary Eclipse Fluorescence Spectrophotometer (Agilent Technologies, Santa Clara, CA, USA). For absorbance normalization, background values measured at 630 nm were subtracted from those measured at 570 nm.

Reactive oxygen species (ROS) were detected, as previously described by Tamma et al. [26]. FaO cells were incubated in a serum-free medium for 1 h before ROS assessment. Then, cells were incubated with dihydrorhodamine-123 (10 µM) (Sigma-Aldrich, Darmstadt, Germany) dissolved in PBS for 30 min at 37 °C with 5% CO_2_ and recovered in a complete medium for 45 min. In the last 30 min of recovery, cells were exposed to scalar concentrations of vehicle (MetOH) or polyphenols. Alternatively, cells were treated with tert-Butyl hydroperoxide (tBHP, 2 mM) (Sigma-Aldrich, Darmstadt, Germany) for 30 min. At last, control or PF-treated FaO cells were lysed in a buffer containing 1% Triton-X-100, 150 mM NaCl, and 25 mM HEPES (pH 7.4). Lysate absorbance was measured at excitation and emission wavelengths of 512 and 530 nm, respectively.

### 2.7. Statistical Analysis

Data were expressed as means ± SD (standard deviation) of at least three independent experiments in triplicate. Statistically significant differences for *p* ≤ 0.05 were assessed by one-way analysis of variance (ANOVA) with Tukey’s post hoc test, using Statistica software, version 7.0 (Stat Soft, Inc. 1984–2004, Tulsa, OK, USA).

Principal component analysis (PCA) of the consistency of sample libraries was assessed by multivariate data analysis with the chemometrics agile tool (CAT) software, version 3.1.2 (http://www.gruppochemiometria.it/index.php/software accessed on 12 December 2023).

## 3. Results

### 3.1. Feature Comparisons of Sanitized and Non-Sanitized BR Plants

**Figure 1:**
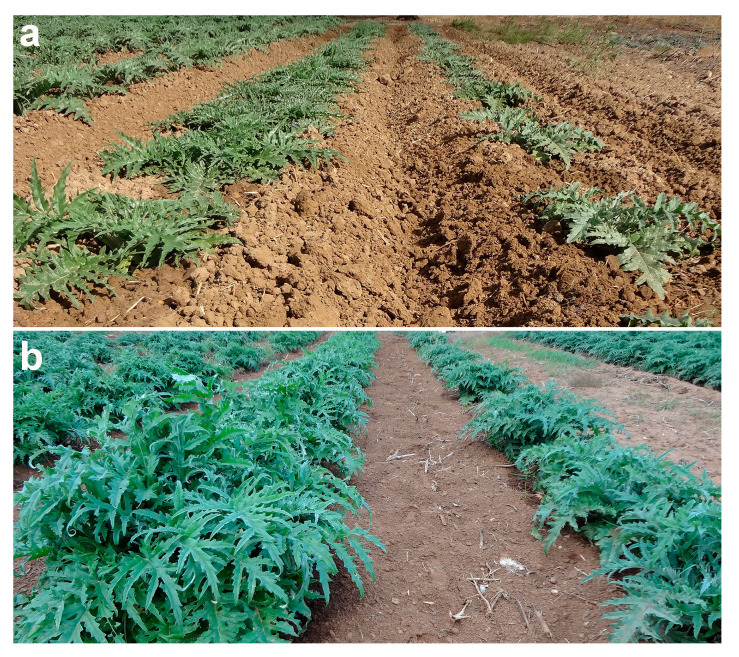
Different development attitudes of BR-S (left row samples) compared to BR-NS plants (right row samples) at (**a**) one year and (**b**) two years after transplanting into the open field.

BR-S artichokes showed a different growth attitude in open-field trials compared to NS artichokes (Figure 1). The superior vigour observed in BR-S plants was more evident starting from the second year after transplanting (Figure 1b).

Although both BR-NS and BR-S plants were asymptomatic, polyprobe hybridization and transcriptome analysis revealed infection by the artichoke Italian latent virus (AILV) and artichoke latent virus (ArLV) in BR-NS plants. None of the other viruses considered were found in the BR-S samples. These results were validated by qRT-PCR [8]. The presence of AILV and ArLV infection in BR-NS plants determines a reduction in plant growth and capitula production [8], whereas BR-S plants showed increased leaf development and larger plant size and a consequent increase in biomass availability from which phenolic compounds may be extracted.

### 3.2. Comparative Analysis of Whole-Transcriptome in Artichoke

Rnaseq analysis was performed on total RNA extracted in triplicate from the BR-S and BR-NS plants. Sequencing experiments on the Illumina platform produced 56 G bases of raw reads with a high-quality score (5.6 over the minimum reference value of 30). Libraries were aligned on the *C. cardunculus* reference genome, resulting in about 80% of total reads mapped, corresponding to 44.6% of total *C. cardunculus* genes. The biological variance between replicates for each condition was calculated using normalized count data showing 97.1% of PC1 vs. 1.9% of PC2 over the total variance in PCA and by separating the BR-S and BR-NS samples.

DESeq2 analysis between BR-S and BR-NS samples resulted in 11,825 significant DEGs (FDR ≤ 0.05), up- (49.2% of DEGs), and downregulated (50.8% of DEGs).

**Figure 2:**
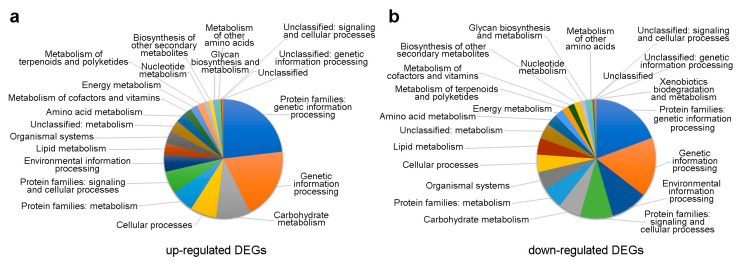
KEGG orthology analysis of (**a**) upregulated and (**b**) downregulated annotated differentially expressed genes (DEGs) in BR-S vs. BR-NS samples using BlastKOALA tool (https://www.kegg.jp/blastkoala/, accessed on 21 June 2023).

Functional characterization analysis classified 2458 (42.2%) upregulated and 2154 (35.8%) downregulated annotated DEGs in many biological pathways (Figure 2). The most represented DEGs were involved in genetic information processing. In this pathway, DEGs were mainly upregulated.

Many overexpressed genes were also observed in carbohydrate metabolism, cellular processes, and protein metabolism. In contrast, the genes involved in signalling, environmental information processing, and organismal systems were mainly downregulated. For the other pathways, an equal number of up and downregulated genes was observed.

**Figure 3:**
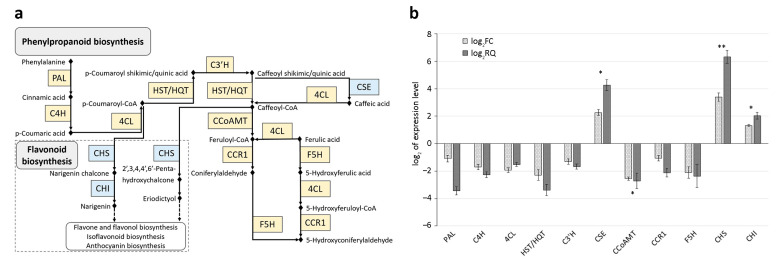
Expression levels of phenylpropanoid and flavonoid biosynthesis DEGs in BR-S compared to BR-NS plants. (**a**) Schematic representation of the biosynthetic pathway with proteins encoded by DESeq2 selected genes. Proteins are reported in boxes along the arrow connecting the substrate to the enzyme product. Light blue boxes indicate proteins encoded by downregulated genes; yellow boxes indicate proteins encoded by upregulated genes. (**b**) RNAseq data validation by quantitative real-time PCR (qPCR) of selected DEGs in S vs. NS BR plants. Columns represent log_2_ of the fold-change of gene expression from RNAseq data (log_2_FC) and values obtained from qPCR analysis (log_2_RQ) calculated as the mean of three biological replicates. The NS condition was used as a non-treated reference plant for gene expression normalization. *Elongation factor 1 alpha* (*EF-1a*) was used as the housekeeping gene for target gene normalization. Bars on the columns indicate the SE for each gene. No statistically significant differences (*p* ≤ 0.05) were observed between the log_2_FC and log_2_RQ values for each gene when performing a Tukey post hoc ANOVA test. (**) double asterisks and (*) single asterisks indicate highly significant and significant differences between genes, respectively, for *p* ≤ 0.05, in a Tukey post hoc ANOVA test. Protein abbreviations: 4CL, 4-coumaric acid: CoA ligase; C3′H, *p*-coumaroyl-shikimic acid/quinic acid 3-hydroxylase; C4H, cinnamic acid 4-hydroxylase; CCoAOMT, caffeoyl-CoA *O*-methyltransferase; CCR1, cinnamoyl-CoA reductase 1; CHI, chalcone isomerase; CHS, chalcone synthase; CSE, caffeoyl shikimic acid esterase; F5H, ferulic acid 5-hydroxylase; HST/HQT, hydroxycinnamoyl-CoA: shikimic/quinic acid hydroxycinnamoyltransferase; PAL, phenylalanine ammonia-lyase.

Functional analysis highlighted 31 and 35 KO entries involved in the biosynthesis of other secondary metabolites, characterized by upregulated and downregulated DEGs, respectively. Among them, genes with |log_2_FC| ≥ 1 were considered to reconstruct a complete pathway module (Figure 3a and Table 1).

Results from KEGG analysis (https://www.kegg.jp/blastkoala/, accessed on 21 June 2023) showed that the genes involved in the phenylpropanoid biosynthesis were mainly downregulated (Figure 3), except for the upregulation of the *Caffeoyl shikimic acid esterase* (*CSE*). The complete module observed in Figure 3a refers to monolignol biosynthesis derived from the phenylpropanoid pathway and is based on the condensation of molecules that have phenylalanine as the basic precursor molecule. The first step is the conversion of phenylalanine to cinnamic acid by PAL. Cinnamic acid is then converted to *p*-coumaric acid by the enzyme cinnamate 4-hydroxylase (C4H) and subsequently transformed to *p*-coumaroyl-CoA by the action of 4-coumaroyl-CoA ligase (4CL). The next step involves the action of a series of enzymes, including HST/HQT, *p*-coumaroyl-shikimic acid/quinic acid 3-hydroxylase (C3′H), caffeoyl-CoA *O*-methyltransferase (CCoAMT), and cinnamoyl-CoA reductase (CCR1), which convert *p*-coumaroyl-CoA to coniferaldehyde. This last molecule, together with the coniferyl alcohol, sinapyl aldehyde, and sinapyl alcohol produced through parallel reactions, serve as precursors for the polymerization of lignin in the cell wall. Monolignols undergo oxidative coupling reactions mediated by peroxidases and laccases to form lignin polymers, which are then deposited in the plant cell wall.

The biosynthesis of monolignols requires the coordination of multiple enzymatic reactions to ensure the proper deposition of lignin in the plant cell wall, such as CSE, which catalyses the conversion of caffeoyl shikimic acid to caffeic acid, a phenolic compound with a crucial role in the production of lignin.

The phenylpropanoid pathway also provides basic precursors for the synthesis of other secondary metabolites in plants. In fact, after the production of *p*-Coumaroyl-CoA and Caffeoyl-CoA by the 4CL enzyme, CHS catalyses their conversion to various chalcone molecules, like narigenin chalcone and 2′,3,4,4′,6′-Penta-hydroxychalcone, which are subsequently converted to flavanone molecules, like narigenin and eriodictyol, respectively, by a series of enzymatic reactions, including CHI. Flavanone molecules represent the substrates for flavone, flavonol, isoflavonoid, and anthocyanin biosynthesis. Genes that encode proteins related to the flavonoid pathway (Figure 3) were upregulated in BR-S plants.

The selected DEGs were validated by qPCR using artichoke *EF-1a* as the housekeeping gene (HK). For this purpose, specific primers were synthesized (Table S1) to amplify cDNAs obtained from RNA preparations of BR-S and BR-NS samples in three biological replicates. PCR efficiency ranged from 91.8 to 109.3% with a regression coefficient (R^2^) of 0.98.

QPCR confirmed the downregulation (log_2_RQ ≥ 1) of all DEGs (*PAL*, *C4H*, *4CL*, *HST/HQT*, *C3′H*, *CCoAMT*, *CCR1*, and *F5H*) involved in the phenylpropanoid biosynthesis pathway in BR-S plants compared to BR-NS samples, and the upregulation of the *CSE* gene (Figure 3b). *CHS* and *CHI* genes of the flavonoid biosynthesis pathway were also upregulated (Figure 3b).

### 3.3. Polyphenol Profile of BR-NS and BR-S Plants

**Figure 4:**
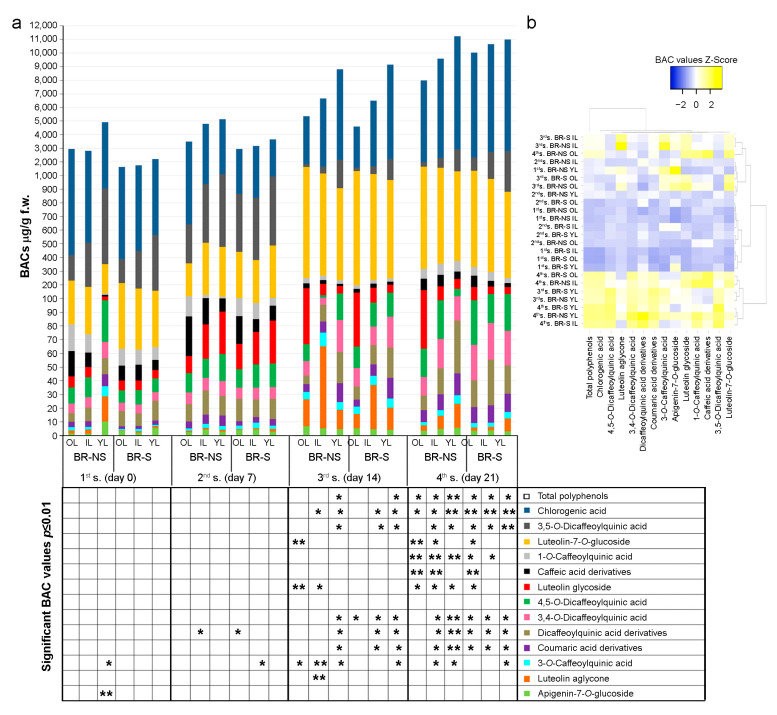
HPLC-DAD time-course quantification of BACs over total polyphenol content in BR-NS and BR-S plants. (**a**) Histogram showing the amount of each compound in weekly collected samples taken from old (OL), intermediate (IL), and young (YL) leaves during May 2022, for a total of four samplings (s.). (**) double asterisks and (*) single asterisk indicate highly significant and significant differences, respectively, for *p* ≤ 0.01, in a Tukey post hoc factorial ANOVA test for each compound analyzed in the time of sampling considered, sanitized conditions, and leaf collected as factors. (**b**) Heatmap of BAC values obtained by clustering compounds and time of collection using the Euclidean distance measurement method.

Time-course analysis of total polyphenol accumulation between the S and NS samples revealed a significant increase in total chemical compounds over the time of the experiment (Figure 4).

In particular, the highest polyphenol content in fresh-weight tissue (f.w.) (over 100 mg/g) was recorded in the YL of BR-NS and BR-S plants, with significant values in the 3rd and 4th samplings. Chlorogenic acid was the most accumulated compound, with highly significant values (8 mg/g f.w.) and a 3.9-fold increase between the 1st and 4th sampling in all BR-S plants and YL plants of BR-NS. In the other NS plants of the 4th sampling, the accumulation of chlorogenic acid was always significant, although with lower values (6.6 mg/g f.w.) compared to BR-S plants, as well as in the YL and IL of the 3rd sampling collected from BR-S and BR-NS artichokes.

Even at lower concentrations, the MCQ as 1-*O*-caffeoylquinic acid and the caffeic derivative compounds showed a significant increase in the 4th sampling of 5.3-fold compared to the 1st sampling. The highest concentration was recorded in BR-NS plants and in OL of BR-S plants, with a mean of 75 μg/g f.w. A significant accumulation (10 μg/g f.w.) of 3-*O*-caffeoylquinic acid was detected in the IL of BR-NS collected during the 3rd sampling, whereas a slightly lower accumulation of this compound (5.6 μg/g f.w.) was observed in other samples, especially in those collected during the 3rd and 4th sampling.

Among the DCQ, 3,5-*O*-dicaffeoylquinic acid was the most represented, which showed a significant accumulation (1.2 mg/g f.w.) in the 3rd and 4th samplings, mainly in the YL of BR-S plants corresponding to a 4.7-fold increase between the 1st and 4th sampling times. No differential accumulation was observed for 4,5-*O*-dicaffeoylquinc acid, while 3,4-*O*-dicaffeoylquinc acid and the other DCQ acid derivatives showed a similar profile and accumulated at high levels (35 μg/g f.w.) in the YL of the BR-NS sample of the 4th sampling. A slightly lower accumulation (24 μg/g f.w.) was observed in BR-S samples at the 3rd and 4th time of samplings and in YL and IL collected from BR-NS during the 3rd and 4th time of samplings, respectively. An identical profile was recorded for the coumaric acid derivatives (mean value 13 μg/g f.w), which showed an overall increase of 3-fold between the 1st and 4th sampling time.

Luteolin-7-*O*-glucoside showed a highly significant accumulation (1.3 mg/g f.w.) in OL of BR-NS at the 3rd and 4th samplings i.e., 8.6-fold higher compared to the 1st sampling. A significant content (1.1 mg/g f.w.) of this compound was also observed at the 4th sampling in the IL of BR-NS and in the OL of BR-S. Although at lower values, a similar profile was recorded for luteolin glycoside, which showed an increase of up to 90 μg/g f.w. in the 4th sampling compared to the 1st sampling, corresponding to an 11-fold increase. Lastly, among the flavonoids, luteolin aglycone showed significant accumulation (60 μg/g f.w.) only in the IL of BR-NS at the 3rd sampling, i.e., 6-fold higher than the mean value for this compound. Similarly, apigenin-7-*O*-glucoside showed a significant accumulation (10 μg/g f.w.) only in the YL of BR-NS plants at the 1st sampling, with an increase of 2.6-fold compared to the other samples.

A heatmap was used to summarize the results from polyphenol time-course quantification in BR-NS and BR-S plants. BACs extracted from leaves collected during the 4th sampling, except for BR-NS OL, clustered together with BR-S and BR-NS YL from the 3rd sampling. BR-NS OL collected in the 4th sampling showed lower total polyphenol values, much more like those observed in BR-S and BR-NS IL of the 3rd sampling. Lower BAC values were observed in BR-S and BR-NS OL of the 3rd sampling, more like those of YL of BR-NS plants collected in the 1st and 2nd samplings and of BR-NS IL of the 2nd sampling. The remaining plants of the 2nd sampling clustered with the BAC values obtained from the IL and OL BR-NS plants of the 1st sampling, while a separate cluster was observed for the leaves taken from the BR-S plants of the 1st sampling.

**Figure 5:**
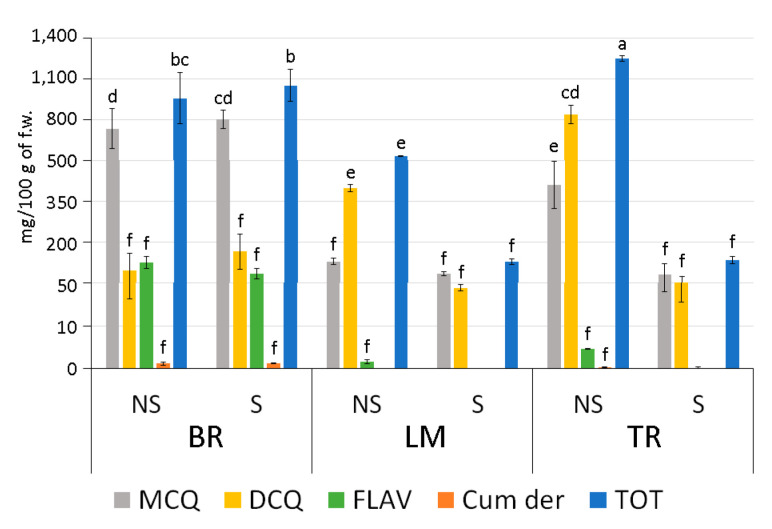
In silico comparison of mono- (MCQ), di- (DCQ) caffeoylquinic acids, flavonoids (FLAV), coumaric derivatives (Cum dev), and total polyphenol (TOT) content in 100 g of fresh leaf material (f.w.) from NS and S artichokes of Brindisino (BR), Locale di Mola (LM), and Troianella (TR) ecotypes. Letters indicate statistically significant differences for *p* ≤ 0.05, performing a Tukey post hoc ANOVA test.

Polyphenol profiles of BR-S and NS samples were compared in silico to those obtained from two late-flowering artichokes, Apulian ecotypes Locale di Mola (LM) and Troianella (TR), analysed in previous studies [12]. Late-flowering ecotypes showed a greater accumulation of total polyphenols in NS condition compared to S plants, whereas the BR ecotype did not show significant variations between S and NS plants (Figure 5). No significant differences were observed in the amount of each polyphenolic fraction in the BR ecotype, while in NS TR and LM plants, a significant increase in DCQ was evident compared to S plants. Moreover, BR plants showed a polyphenol profile particularly rich in MCQ, both in NS and S conditions, while late-flowering ecotypes were richer in DCQ when non-sanitized and showed a similar MCQ and DCQ content consequent to sanitation. In addition, the flavonoid and coumaric derivative contents were reduced in NS late-flowering ecotypes LM and TR compared to S ones. Whereas in the BR ecotype, the same compounds were present at higher concentrations and did not show differences between the NS and S conditions.

### 3.4. Rat Hepatoma FaO Cells Response to Artichoke Leaves Extracts Administration

**Figure 6:**
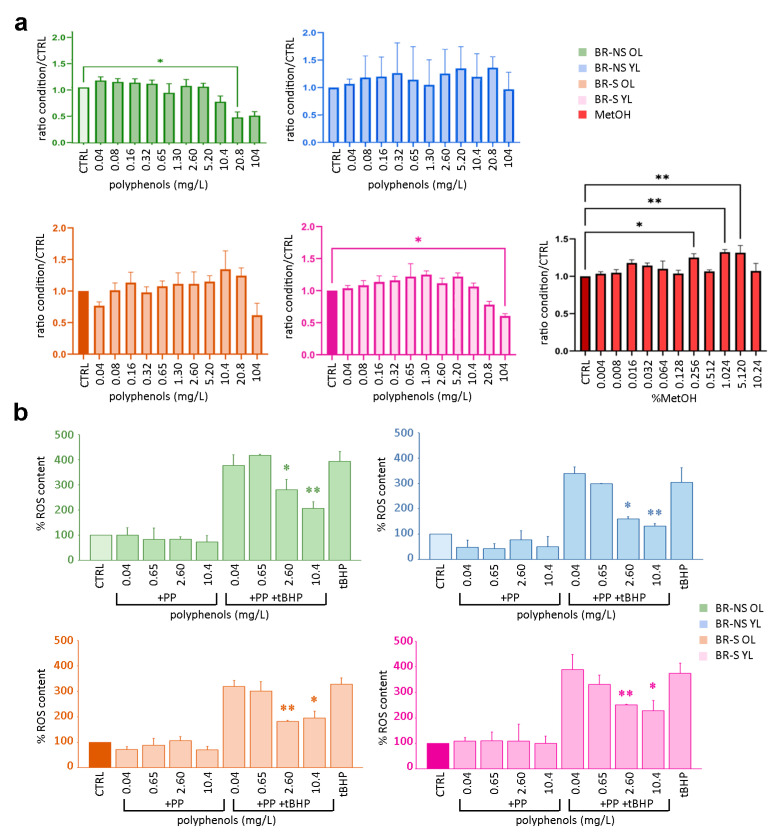
Analysis of rat hepatoma FaO cell response to artichoke extract treatment. (**a**) Integrity of FaO cells exposed to different concentrations of polyphenols resuspended in methanol (MetOH). Extracts were obtained from the old (OL) and young (YL) leaves of BR-NS and BR-S plants. Cytotoxic MetOH effects were evaluated by treating the cells with a scalar dilution of 80% MetOH. (**b**) Dose–response of polyphenol (PP) extracts FaO cells treatment to the accumulation of reactive oxygen species (ROS) in the absence or presence of an oxidative agent (+tBHP). Values represent the means ± standard errors of three independent experiments. CTRL represents the not-treated control used as a reference. (**) double asterisks and (*) single asterisk indicate highly significant and significant differences, respectively, for *p* ≤ 0.05, in a one-way ANOVA test.

A growing number of bioactive phytocompounds known for their beneficial and antioxidant effects on human health could promote cytotoxicity by inducing ROS production [27]. Hence, we evaluated the in vitro cell integrity and antioxidant response of rat hepatoma FaO cells exposed to scalar concentrations of artichoke polyphenols. FaO cells were incubated with increasing scalar concentrations (from 0.04 mg/L to 104 mg/L) of artichoke leaf extracts obtained from the 4th sampling, characterized by the higher amount of total polyphenol collected (Figure 6a). Compared to the control FaO cells exposed to MetOH/water (50:50, *v*/*v*) (used as a solvent for polyphenol extracts at no cytotoxic concentrations ranging from 0.04 to 10.4%, Figure 6a *red histograms*), only BR-NS OL caused a significant decrease in cell integrity starting from the concentration of 20.8 mg/L (Figure 6a *green histograms*). A cell integrity reduction of about 50% was detected in FaO cells incubated with BR-NS OL or BR-S YL extracts at a concentration of 104 mg/L (Figure 6a *green and pink histograms*, respectively).

Next, the antioxidant activity exerted by the methanolic extracts of artichoke leaves was assessed in FaO cells exposed to concentrations of polyphenols ranging from 0.04 to 10.4 mg/L (Figure 6b). The antioxidant activity was expressed as the percentage of induced ROS levels of the artichoke methanolic extracts compared with the solvent alone (MetOH). Compared to the positive control [FaO cells treated with 2 mM of the oxidant tert-Butyl Hydroperoxide (tBHP)] [26], cells exposed to polyphenols derived from NS and S leaves showed a significant decrease in ROS content (−57%) when incubated with 10.4 mg/L of extract obtained from BR-NS YL (Figure 6b; *light blue histogram*). Moreover, cells exposed to 2.6 mg/L and 10.4 mg/L of polyphenols obtained from BR-NS YL and BR-NS OL displayed a 50% reduction in ROS levels (Figure 6b *light blue and green histograms,* respectively). A 30% ROS content reduction was also observed when the cells were incubated with 2.6 mg/L of polyphenols extracted from BR-NS OL (Figure 6b *green histogram*). In addition, ROS levels assessed by using extracts from S plants showed a significant reduction when 2.6 mg/L and 10.4 mg/L from OL were tested (−45% and −41%, respectively) (Figure 6b *orange and pink histograms*, respectively). Similar results were obtained by using BR-S YL extracts (−33% and −40%, with 2.6 mg/L and 10.4 mg/L, respectively) (Figure 6b *pink histogram*).

Taken together, these results demonstrate that 10 mg/L polyphenols extracted from OL or YL of BR-S artichoke plants have sufficient antioxidant activity without compromising cellular integrity.

**Figure 7:**
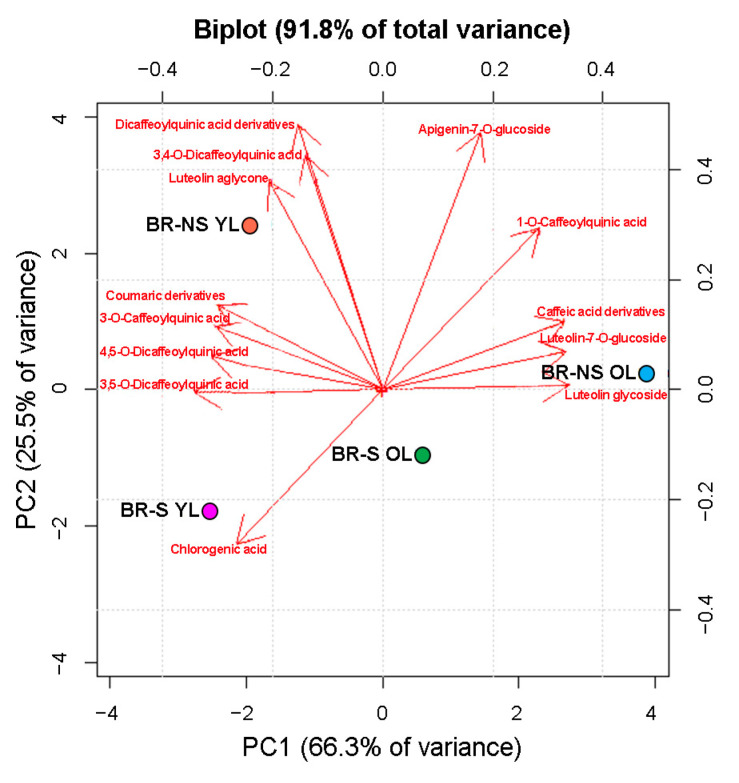
Principal component (PC) analysis of polyphenol content in sanitized (S) and non-sanitized (NS) Brindisino (BR) samples collected from old (OL) and young leaves (YL).

PCA was used to investigate the main difference in total polyphenol composition between BR-S and NS extracts. Biplot analysis showed that 91.8% of the total variance was divided into 66.3% and 25.5% between PC1 and PC2, respectively (Figure 7).

PCA showed that BR-S extracts obtained from YL and OL were very similar to each other. On the contrary, the extract obtained from YL of BR-NS was different from that obtained from OL. The composition of BR-NS OL extract was significantly different from the other extracts because of the higher amount of 1-*O*-caffeoylquinic acid, caffeic acid derivatives, luteolin glycoside, and luteolin-7-*O*-glucoside, and the reduced content of chlorogenic acid, 3,5-*O*-dicaffeoylquinic acid, and coumaric derivatives compared to the other samples. The extracts obtained from the YL of the BR-NS samples were characterized by the significant presence of luteolin aglycone and coumaric derivatives. On the other hand, the composition of extracts from BR-S plants showed no significantly higher amount of specific polyphenol compounds and reduced content of luteolin aglycone in OL, and 1-*O*-caffeoylquinic acid, caffeic acid derivatives, and apigenin-7-*O*-glucoside in YL.

Furthermore, a similar quantity of apigenin-7-*O*-glucoside was observed between BR-NS samples, whereas YL and OL extracts collected from BR-S plants both showed a higher chlorogenic acid content and a lower DCQ acid derivative content.

### 3.5. Polyphenols Green Extraction

**Figure 8:**
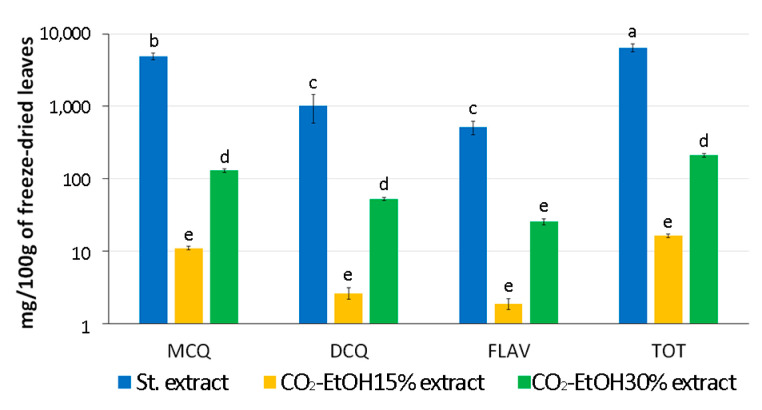
HPLC-DAD quantification of mono- (MCQ), di- (DCQ) caffeoylquinic acids, and flavonoids (FLAV) over total polyphenol (TOT) content extracted from sanitized Brindisino artichokes. Standard extraction (St. extract) from 100 g of freeze-dried leaves was compared to CO_2_ supercritical fluid extraction with co-solvent ethanol (CO_2_-EtOH extract) at concentrations of 15% and 30%. Letters indicate statistically significant differences for *p* ≤ 0.05 performing a Tukey post hoc ANOVA test.

To reduce environmental contamination due to the use of substances such as methanol in the polyphenol extraction processes, leaves collected from BR-S artichoke plants were subjected to SFE with co-solvent EtOH at concentrations of 15% and 30%. The results obtained were compared with those obtained by standard extraction, highlighting that the CO_2_ extraction protocol tested allowed the isolation of a smaller quantity of phenolic compounds from the same quantity of freeze-dried plant material (Figure 8).

A 400-fold reduction in total polyphenols was observed in the presence of 15% EtOH and 30-fold with 30% EtOH, compared to the standard extraction technique. In addition to the significant decrease in the quantity of MCQ, DCQ, and flavonoids, the co-extraction of impure substances and chlorophyll was observed in the extracts obtained with supercritical CO_2_, while coumarin-derived compounds were absent. To selectively extract only the bioactive complexes of interest and to avoid further purification procedures of the extract, it will be necessary to proceed with further modifications to the protocol by regulating the temperature (T) and pressure (P) of the extraction system and, therefore, the density of the CO_2_ introduced. The definition of the optimal extraction protocol would allow for the scale-up of the extraction process and its application at an industrial level.

## 4. Discussion

Plant vigour refers to the overall health, growth, and vitality conditions. It mostly depends on genetic factors and environmental growing conditions. Meristem-tip-culture and thermotherapy applied to produce virus-free germplasm could also influence plant vigour when proper protocols are followed to ensure higher agronomic performance of S plants vs. NS counterparts [8,12]. Similarly, during the acclimatization process characterized by a gradual adaptation to the outdoor environment, appropriate watering and fertilization practices contribute to the robustness of the plant, resulting in enhanced growth of S artichokes compared to NS artichokes.

Plant viral infections induce disorders in gene expression and metabolic processes, resulting in the appearance of strong disease symptoms such as leaf mosaic, yellowing, necrosis, and morphological changes in plant and/or fruit structures [28], but also no symptoms at all. In asymptomatic infected plants, only a mild stunted growth may be detectable by direct comparison with the virus-free counterpart, as in the case seen with BR-NS and BR-S (Figure 1). In addition to morphological and chromatic changes, the virus alters host cellular processes to facilitate replication and plant colonization, and the host plant must adapt to the metabolic changes induced by viral infection (Figure 2). This manipulation includes redirecting host resources and altering metabolic pathways and energy requirements to meet the demands of viral replication, as observed in this study (Figure 2) and in other studies [28]. This shift in energy metabolism manifests with the accumulation of carbohydrates, amino acids, and other metabolites, like phenylpropanoids, to produce the basal compounds for the biosynthesis of monolignols, the building blocks of lignin. Lignin is an important component of plant cell walls, providing strength and rigidity during plant development. It is the last process of secondary cell wall biosynthesis but is also known to counteract pathogenic diseases. Therefore, reduced expression of genes related to these mechanisms is expected in artichoke plants sanitized from viral infections. Nevertheless, the regulation of monolignol biosynthesis involves the action of various transcription factors and signalling molecules that respond to developmental and environmental stimuli through various complex mechanisms, which are still poorly described in plant science [29].

Similarly, plants increase the synthesis of polyphenols also in response to abiotic stresses [30]. Whether the sanitation process through meristem-tip-culture and thermotherapy could be listed as abiotic stress or not is unknown. Abiotic stresses cause alterations in the biochemical and physiological processes of plants due to the rapid changes in cellular redox homeostasis and the generation of excessive reactive oxygen species (ROS), which result in peroxidation and destabilization of cellular membranes. These stresses reduce plant growth and yield [31]. To restore normal plant functions, more phenolic compounds were synthesized [32], resulting in the reduction of cell membrane peroxidation [33]. The biosynthesis of phenolic compounds under stressful conditions was reported to be characterized by the upregulation of the transcript levels of key genes like *PAL*, *C4H*, *4CL*, *CHS*, and *CHI* (Figure 3) [30]. The downregulation of *PAL*, *C4H*, and *4CL* observed in this study (Figure 3) demonstrates the absence of stressful conditions in BR-S artichokes compared to BR-NS ones. Although, a significant upregulation of *CHS* and *CHI* genes (Figure 3), implicated in flavonoid biosynthesis, was observed. Flavonoids are involved in plant protection against pathogens in response to environmental signals [34] and in plant growth in response to developmental signals. Moreover, flavonoids reduce oxidative damage caused by reactive oxygen species (ROS), which influence plant metabolism and physiology [35]. Flavonoids can be divided into two groups: “preformed”, synthesized during the general development of plants, like in S artichokes, and “induced”, synthesized after stress based on their mode of action [36], like in BR-NS artichokes.

The involvement of phenylpropanoids in many stimuli derives from the enormous variability of chemical structures that are generated from the phenylalanine skeleton, such as flavonoids, monolignols, phenolic acids, stilbenes, phytoalexins, and coumarins, which are directly and indirectly involved in plant development and disease response [37,38].

However, polyphenols are also produced when the plant grows under optimal conditions, although at lower levels compared to when the plant is under stress, playing a crucial role in the development of the plant by regulating signal transduction, hormone signalling, photosynthetic activity, germination, cell division, and reproduction rate.

Similar values of total polyphenols between BR-S and BR-NS plants at the same sampling time (Figure 4) suggest the presence of epigenetic factors conserved in BR-S plants after in vitro culture and/or activation during the sanitation protocol. The ability of plants to sense, respond, and remember environmental stimuli is essential for their survival. Plastic plant development can occur in response to anticipated changes in the environment, such as stressful events, exposure to pathogen infection, and in vitro culture propagation [39,40,41]. The possibility of memorizing stresses, such as epigenetic modification of the plant genome, may later prime the plant to respond to such stimuli or be durably primed with functional states of adaptive immunity. This immunity contributes to a non-specific protective effect, which could also be seen in a higher baseline level of polyphenols in BR-S plants compared to BR-NS plants without significant activation of gene transcription. Similar observations were made in tomato ecotypes tolerant to viral infection, characterized by mild symptoms and low transcriptome modulation compared to more susceptible plants [42].

Polyphenolic compounds have been shown in animal studies to have positive health effects due to their antioxidant activity, metabolism regulation, and cancer prevention [43].

Polyphenol treatment of FaO cells, a hepatocyte cell line, showed reduced cell integrity in a dose-dependent manner based on the polyphenol composition of each extract (Figure 6). The higher content of 1-*O*-caffeoylquinic acid, caffeic acid derivatives, luteolin glycoside, and luteolin-7-*O*-glucoside, and the reduced content of chlorogenic acid, 3,5-*O*-dicaffeoylquinic acid, and coumaric derivatives in the polyphenol profile of BR-NS OL samples, compared to the other extracts, seems to be cytotoxic at extract concentrations of 12 mg/L. FaO cells treated with 90 mg/L polyphenol obtained from the other samples tested did not show cytotoxic effects. Therefore, we propose that the higher concentration of specific compounds in BR-NS artichokes compared to BR-S might be attributed to the viral infection detected by RNAseq analysis and qPCR.

A polyphenol concentration-dependent effect was also observed by analysing the oxidative cell response when different quantities of the extracts were supplied (Figure 6b). A higher antioxidant activity at a safe concentration range for cells could be obtained by using 10 mg/L of the extract with a polyphenol profile more similar to that of BR-NS YL and BR-S OL, characterized by 0.65 mg/L of 1-*O*-caffeoylquinic acid, 0.04 mg/L of 3-*O*-caffeoylquinic acid, 76 mg/L of chlorogenic acid, 67 mg/L of caffeic acid derivatives, 0.26 mg/L of 3,4-*O*-dicaffeoylquinic acid, 11 mg/L of 3,5-*O*-Dicaffeoylquinic acid, 37 mg/L of 4,5-*O*-dicaffeoylquinic acid, 28 mg/L of dicaffeoylquinic acid derivatives, 0.04 mg/L of apigenin-7-*O*-glucoside, 0.05 mg/L of luteolin aglycone, 0.74 mg/L of luteolin glycoside, 9.5 mg/L of luteolin-7-*O*-glucoside, 0.13 mg/L of coumaric derivatives in 100 mg/L of total polyphenols. Even if the extracts obtained from BR-NS YL have a more significant effect on the cells in terms of ROS reduction, NS plants cannot guarantee an extract with a defined and constant profile, being dependent on the virus species and infection process. For this reason, S artichokes, grown under controlled conditions, can provide higher quality plant material, which can ensure a more defined quantity of extracted compounds.

However, the final composition of polyphenol extracts must also consider the final MetOH concentration since, as reported in the results of this work, even quantities equal to 1% could be harmful to cell integrity (Figure 6a). Therefore, SCFE was evaluated to propose a sustainable green extraction method. This technique is largely recognized as a safe and green approach, and at the same time, it also allows the recovery of high quantities of molecules of interest [44].

SCFE requires moderate working temperature and pressure for the separation of polyphenols from plant materials. It was also reported that this approach preserves the functional properties of the compounds; therefore, a reduced cytotoxicity effect and a higher antioxidant activity were expected when BR-S extracts in EtOH solution were used, compared to MetOH. The use of co-solvents, like EtOH, is recommended to increase the amount of polyphenol extracted [44,45]. Moreover, the highest antioxidant ability was reported for the extract obtained under operational conditions of 50 °C and 300 bar [46], as performed in this study. The parameters used for the SCFE of polyphenols from artichoke leaves allowed the extraction of only a reduced fraction of these compounds compared to what was obtained through a standard extraction (Figure 8). Increasing the amount of EtOH from 15% to 30% increased the amounts of extracted polyphenols; however, new experiments will certainly be necessary to better set up the SCFE parameters. For example, the use of 40% EtOH-MetOH mixture was reported optimal for guaraná (*Paullinia cupana*) seeds, from which can be obtained a total phenolics content of 100 pyrogallol equivalent per gram of guaraná seeds [47]. Therefore, the only limiting factor in using the SCFE technique is the optimization of operating parameters on the selectivity and yield of the target product.

## 5. Conclusions and Future Perspectives

Virus infection in global artichokes compromises the overall quality and productivity of crops. The use of virus-free plant material obtained through an in vitro sanitation protocol and multiplied in commercial nurseries conforms to current EU Directives 93/61/CEE and 93/62/CEE, and Plant Health Regulations (EU) 2016/2031 and (EU) 2017/625, [12,48,49], guarantees high-quality production and prevents the spread of pathogens. Viral infections strongly influence the polyphenol composition of plant extracts due to their involvement in plant defence mechanisms. Interactions between virus and polyphenol composition can be complex, but polyphenols also have antioxidant properties and, in the absence of virus infection, help the plants counteract oxidative stress and contribute to overall cellular health. The presence of viral infection in plants prior to sanitation may have activated the constitutive production of polyphenols. Therefore, in sanitized virus-free plants, polyphenols may be present at higher levels via an epigenetic mechanism and exhibit enhanced bioactivity even in the absence of viral stressors.

To our knowledge, this work is the first study reporting the secondary metabolite profile of an early-flowering Apulian artichoke ecotype sanitized and characterized by stable polyphenol production, allowing the prediction of leaf extract composition. The prediction of the most favourable polyphenol extraction time makes BR-S artichokes highly valuable for medicinal purposes and not only for fresh markets.

Further work is needed to assess the intricate interplay existing among plant stress responses, polyphenol production, and polyphenol bioactivity profile.

## Figures and Tables

**Table 1:** DEGs between the BR-S and BR-NS conditions were involved in the biosynthesis of secondary metabolites.

Gene Name	GeneID ^1^	TranscriptID ^2^	KO ^3^	EC N. ^4^	Genome Position
*PAL: Phenylalanine ammonia-lyase*	*Ccrd_014855*	KVI06790	K10775	4.3.1.24	scaffold_5442:7052:11961:-1
*C4H: Cinnamic acid 4-hydroxylase*	*Ccrd_003800*	KVH94141	K00487	1.14.14.91	scaffold_304:130579:133751:1
*4CL: 4-coumaric acid:CoA ligase*	*Ccrd_021802*	KVI00019	K01904	6.2.1.12	scaffold_8:949649:950835:-1
*HST/HQT: Hydroxycinnamoyl-CoA:shikimic/* *quinic acid hydroxycinnamoyltransferase*	*Ccrd_022724*	KVH99042	K13065	2.3.1.133	scaffold_84:128605:129897:1
C3′H: *p-coumaroyl-shikimic acid/quinic acid 3-hydroxylase*	*Ccrd_018265*	KVI03429	K09754	1.14.14.96	scaffold_679:156406:161089:-1
*CSE: Caffeoyl shikimic acid esterase*	*Ccrd_011837*	KVI09726	K18368	3.1.1-	scaffold_21:58721:62131:-1
*CCoAMT: Caffeoyl-CoA O-methyltransferase*	*Ccrd_019031*	KVI02677	K00588	2.1.1.104	scaffold_364:132763:150464:1
*CCR1: Cinnamoyl-CoA reductase 1*	*Ccrd_015577*	KVI06073	K09753	1.2.1.44	scaffold_150:97256:103674:-1
*F5H: Ferulic acid 5-hydroxylase*	*Ccrd_005646*	KVH92322	K09755	1.14.-,-	scaffold_307:82455:84491:1
*CHS: Chalcone synthase*	*Ccrd_012767*	KVI08856	K00660	2.3.1.74	scaffold_4274:28152:29501:1
*CHI: chalcone isomerase*	*Ccrd_014697*	KVI06946	K01859	5.5.1.6	scaffold_915:82010:92443:-1

^1^ Gene identification number. ^2^ Relative gene identification number transcript. ^3^ KEGG orthology. ^4^ Enzyme Commission number.

## Data Availability

Sequencing data are available from NCBI Bioproject n. PRJNA1034968.

## Supplementary Materials

The following supporting information can be downloaded at https://www.mdpi.com/article/10.3390/antiox13070852/s1, Table S1: List of primers used for validation of transcriptome results by qPCR.
